# Antibiotic resistance in *Neisseria gonorrhoea* and treatment outcomes of gonococcal urethritis suspected patients in two large hospitals in Bhutan, 2015

**DOI:** 10.1371/journal.pone.0201721

**Published:** 2018-08-01

**Authors:** Tshokey Tshokey, Thupten Tshering, Ambika Rani Pradhan, Deepika Adhikari, Ragunath Sharma, Kiran Gurung, Tshewang Dorji, Sangay Wangmo, Ugen Dorji, Kinley Wangdi

**Affiliations:** 1 Department of Laboratory Medicine, Jigme Dorji Wangchuck National Referral Hospital (JDWNRH), Thimphu, Bhutan; 2 Department of Pharmacy, JDWNRH, Thimphu, Bhutan; 3 Department of Dermatology, JDWNRH, Thimphu, Bhutan; 4 Essential Medicine and Technology Division, Ministry of Health, Thimphu, Bhutan; 5 Phuentsholing General Hospital, Phuentsholing, Bhutan; Emory University School of Medicine, UNITED STATES

## Abstract

**Introduction:**

Gonorrhea is a major sexually transmitted infection (STI) globally with increasing trends. Despite limited data, gonorrhea remains an important public health problem in Bhutan.

**Methods:**

A descriptive study was carried out in two Bhutanese hospitals; Jigme Dorji Wangchuck National Referral Hospital and Phuentsholing General Hospital in 2015. Patients suspected of gonococcal urethritis were sampled, treated and followed up at two weeks. Gonococcal isolates were identified and tested for antibiotic susceptibility by the Calibrated Dichotomous Sensitivity Test (CDS) method.

**Results:**

Of the 524 patients, 2.3% (12) were females. Most (46.6%) patients belonged to the 26–35 years age group. About 58% were lost to follow up; 62% (277) of males and all (12) females. *N*. *gonorrhoea* was positive in 76% (398) of microscopy and 73.1% (383) by culture. Resistance against ciprofloxacin, penicillin, tetracycline and nalidixic acid were 85.1%, 99.2%, 84.8% and 99.7% respectively. Nearly all the isolates were sensitive to cefpodoxime, ceftriaxone and azithromycin. Sixty-seven percent (350) were treated with injection ceftriaxone alone, 32% (169) with ceftriaxone and oral doxycycline and 1% (5) with ceftriaxone, doxycycline and metronidazole. Probable treatment failure was seen only in one patient (0.5%).

**Conclusions:**

Gonococcal resistance to currently used antibiotics was low and there was a high clinical cure rate. Compliance to treatment guidelines need reinforcement addressing antibiotic regimen, tracing sexual partners and addressing the social stigma. National STI programs should be more women-friendly for effective management, prevention and control of STIs. Laboratories must adopt more reliable susceptibility testing methods, the Minimum Inhibition Concentration method.

## Introduction

Gonorrhea is a major sexually transmitted infection (STI) caused by the bacterium *Neisseria gonorrhoeae*, with an estimated 78 (53–110) million global cases in 2012[1]. Prevalence and incidence estimates vary widely by gender and region of the world. Adding to the problem of increasing cases, there is emerging antibiotic resistance amongst *N*. *gonorrhoea* complicating treatment and risking treatment failure [2]. Antibiotic resistance in *N*. *gonorrhoea* has followed the same pattern over years, with the introduction of each new therapeutic agent followed by the development of resistance within a few years [3]. Resistance to penicillin and tetracycline emerged in Asia as early as 1970 and to fluoroquinolones in the mid-1990s, resistance to both now becoming widespread globally. Resistance to ceftriaxone has now been reported from all regions of the World Health Organization (WHO) [4]. Amongst countries of the WHO Southeast Asia region (SEAR), resistance to ceftriaxone was heterogeneous ranging from 18% in Myanmar (18 isolates) to 3.9% in India with no reports of resistance in Nepal (7 isolates) and Sri Lanka (75 isolates). Resistance to ciprofloxacin increased from 9% in 1997 to 87% in 2006 in Bangladesh [5] and from 3.4% in 1996 to 83.3% in 2008 in India [6]. In 2012, gonococcal resistance against ceftriaxone in Bhutan was between 0.2% to 2.2% [4]. A recent study reported high resistance to nalidixic acid 96.3%, ciprofloxacin 89.3%, penicillin G 87.8% and tetracycline 72.9%. However, resistance against other antibiotics were low including azithromycin2.0%, cefpodoxime 1.9%, spectinomycin 0.8%, and ceftriaxone 0.2% [7].

The WHO provide six treatment options for specific conditions caused by *N*. *gonorrhoeae* emphasizing that the choice of therapy (both dual and single) should be best determined by local resistance data. Where local data is lacking, dual therapy is preferred over monotherapy for genital or anogenital infections [8]. The Centre for Disease Control and Prevention (CDC) also recommends dual therapy for uncomplicated gonococcal infection of the urethra, cervix and rectum; a single dose of ceftriaxone 250 mg intramuscularly (IM) with a single dose of azithromycin 1 gram orally given together on the same day [9]. A recent outbreak of high-level azithromycin resistant *N*. *gonorrhoea* was detected in England threatening this front-line therapy [10]. Currently, there are limited treatment outcome studies from Asia with most published literature from the developed countries. A Canadian study detected 17.8% treatment failure with cefixime 400-mg single dose but none in those treated with either ceftriaxone monotherapy or cefixime/ceftriaxone combined with azithromycin or doxycycline [11]. Varying degree of treatment failures have also been reported from Australia [12], Japan [13] and the Netherlands [14].

STIs remain a serious public health problem in Bhutan. The annual health bulletin of the Bhutan Ministry of Health (MoH) reported an increase in the incidence of STIs from 12/10,000 population in 2011 to 92/10,000 population in 2015[15]. Although pelvic inflammatory disease and infertility are the main complications of gonorrhea, there are no data on these in Bhutan. Bhutan continues to use ceftriaxone 250 mg IM injection single dose, monotherapy as the empirical treatment for gonorrhoea since 2006, as outlined in the National Antibiotic Guideline, 2012 [16]. Current antibiotic resistance level against ceftriaxone is acceptable (<5%) to use the drug as a primary drug of choice [17]. However, with no medical colleges in the country and doctors trained in different neighbouring countries, it remains a challenge to implement the uniform adherence to national guidelines without formal orientation of these medical graduates of diverse backgrounds joining the Bhutanese health system. Although the current treatment regimen was initiated more than a decade ago, clinical and microbiological responses to treatment has never been assessed. Therefore, the present study aimed to investigate the antimicrobial susceptibility of *N*. *gonorrhoea* and correlate with treatment outcomes with the empirical treatment of gonorrhea in two large Bhutanese hospitals.

## Materials and methods

### Research design

**Fig 1:**
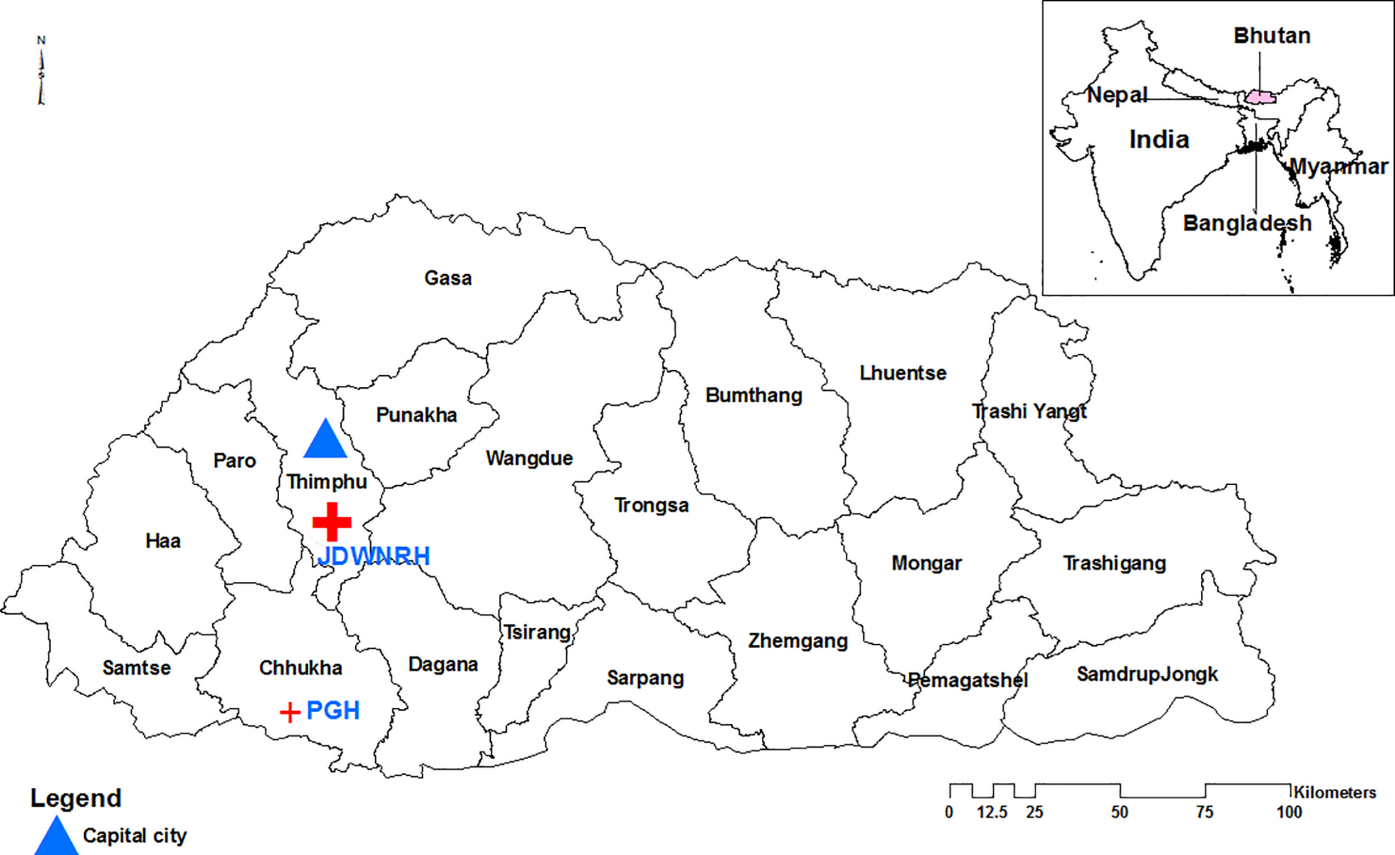
Map of Bhutan showing the study sites. JDWNRH (Jigme Dorji Wangchuck National Referral Hospital; PGH (Phuentsholing General Hospital).

A descriptive study was carried out in two large hospitals in Bhutan; the Jigme Dorji Wangchuck National Referral Hospital (JDWNRH) in Bhutan’s capital city, Thimphu and Phuentsholing General Hospital (PGH) located in the commercial city of Phuentsholing bordering India **(Fig 1)** from January-December 2015. A study protocol and information collection details were developed by reviewing previous literature and adapting to the local setting. Relevant physicians, laboratory and pharmacy staff were briefed on the study before commencement in both the hospitals.

### Study participants and recruitment

All patients suspected of gonococcal urethritis and prescribed the empirical therapy by physicians in the two hospitals were included in the study. From consultation patients were referred to the microbiology laboratory for sampling. After providing informed consent, demographic information, and clinical details and urethral/cervical swabs were collected by laboratory staffs in male patients and by the treating physicians/gynaecologists in female patients. From the laboratory, patients proceeded to the pharmacy unit to receive the prescribed treatment/s. Additional information on present and past treatments and contact phone numbers were collected by pharmacy staff. Patients were followed up after two weeks of treatment through phone calls to elicit the persistence of symptoms. Patients with no symptoms were classified as clinically cured and in the event symptoms persisted, they were called back for clinical re-evaluation and testing. Additional questions on sexual habits were administered during re-evaluation and urethral/cervical swabs collected and re-tested.

### Laboratory testing

The samples were subjected to microscopy by Gram’s stain and microbiological culture using chocolate agar or modified Thayer Martin agar when available. Microscopy was considered positive with Gram-negative intra and extracellular diplococci in direct smears. Isolates from positive cultures were identified as *N*. *gonorrhoeae* with observation of Gram-negative diplococci in Gram stain, positive oxidase and superoxol (catalase) tests supported by API NH commercial kit (Biomerieux, USA). Antibiotic susceptibility was tested using the Calibrated Dichotomous Sensitivity (CDS) method established in both the laboratories. The CDS method is an acceptable method for gonococcal resistance testing in the WHO Gonococcal Antimicrobial Surveillance Programme (WHO GASP), recommended especially in low resource settings and widely used in the WHO South East Asia region (WHO SEAR), including Bhutan. The two laboratories regularly participate in external quality assessment schemes of the WHO GASP, with WHO collaborating centres in India (Apex laboratory, New Delhi) and Australia (SEALS Microbiology, Sydney). The WHO GASP recommends that any decreased susceptibility or resistance identified in extended-spectrum cephalosporins by disc diffusion be verified with quantitative MIC determination [18]. CDS results were interpreted as sensitive (S), resistant (R) or decreased susceptibility (DS) as per the low potency discs (Oxoid, England) and annular radius breakpoints for each antibiotic **(“S1 Table”).** Quality control of CDS method was routinely assured by testing with standard *N*. *gonorrhoeae* strain of known sensitivity supplied by reference laboratory in Sydney.

### Statistical analysis

Data was entered in Microsoft Excel spreadsheet and analyzed using STATA 12.1. Descriptive statistics were carried out by providing the number and percentage of each of the demographic variables as well as the questions on sexual history and clinical symptoms. Chi-squared test and logistic regression analysis were carried out to find associations and determinants for loss to follow-up with all variables. Bivariate and multivariable logistic regression models for lost to follow was built using backward elimination to identify significant covariates. An alpha level of 0.10 was used to determine which variables remained in the model. A value of p≤ 0.05 was considered significant. All explanatory variables in the multivariable model were tested to ensure there was no multi-collinearity in the final model.

### Ethics statement

Ethical clearance was sought from the Research Ethics Board of Health (REBH), Ministry of Health, Bhutan vide approval number REBH/Approval/2014/020. Interviewers explained the general purpose, benefits, and any risks of the study to each respondent in his or her local language, and respondents had the right to refuse participation in the study at any point. All participant details were anonymized.

## Results

### Descriptive analysis

A total of 524 participants were followed up from January to December 2015 in the two hospitals. Only 2.3% (12) of patients were females. More than half of the participants, 58.0% (304) were lost to follow up leaving only 42% (220) with known treatment outcomes. Most participants belonged to the age groups of 26–35 years (46.6%) and 19–25 years (36.1%). About 87.6% (459) participants were from the JDWNRH and most of the lost to follow up 63.0% (289) was recorded in the JDWNRH. In PGH, only 23.1% (15) were lost to follow up. The commonest age group that was lost to follow up was 26–35 years at 49.3% (150). Sixty-two percent (277) of males and 100% (12) of females were lost to follow up. Private and corporate workers, drivers and civil servants were most likely to be lost to follow up at 56.6% (164), 13.5% (39) and 13.1% (38) respectively. Regarding sexual behaviour, participants who claimed not to engage in casual sex were mostly lost to follow up at 81.4% (249). Similarly, participants who denied multiple sexual partners, history of suffering from STI and having been treated for **STI** were lost to follow up at 59.9% (302), 63.6% (283) and 63.2 (283), respectively (**Table 1**).

**Table 1:** Characteristics of study population and treatment follow-up outcome.

**Characteristics**		**Total (%)**	**Clinical cure (%)**	**Lost to follow up (%)**	**p value**
**Hospitals**	JDWNRH	459 (87.6)	170 (37.0)	289 (63.0)	<0.0001
	PGH	65 (12.4)	50 (76.9)	15 (23.1)	
**Socio- demographic characters**					
Age group (years)	< 19	13 (2.4)	2 (0.9)	11 (3.6)	0.048
	19–25	189 (36.1)	91 (41.4)	98 (32.2)	
	26–35	244 (46.6)	94 (42.7)	150 (49.3)	
	36–45	58 (11.1)	27 (12.3)	31 (10.2)	
	> 45	20 (3.8)	6 (2.7)	14 (4.61)	
Gender*	Male	447 (97.4)	170 (38.0)	277 (62.0)	0.007
	Female	12 (2.6)	0 (0)	12 (100.0)	
Occupation*	Civil servants	57 (11.2)	19 (8.7)	38 (13.1)	0.181
	Private/corporate workers	290 (57.0)	126 (57.5)	164 (56.6)	
	Drivers	66 (13.0)	27 (12.3)	39 (13.5)	
	Students	33 (6.4)	12 (5.5)	21 (7.2)	
	Armed personals	17 (3.3)	11 (5.0)	6 (2.1)	
	Tourist guide	20 (3.9)	9 (4.1)	11 (3.8)	
	Religious body	15 (3.0)	7 (3.2)	8 (2.8)	
	Others	11 (2.2)	8 (3.7)	3 (1.0)	
**Symptoms**					
Urethral discharge	No	8 (1.5)	0 (0)	8 (100.0)	0.023
	Yes	516 (98.5)	220 (42.6)	296 (57.4)	
Dysuria*	No	62 (11.8)	48 (77.4)	14 (22.6)	<0.0001
	Yes	462 (88.2)	172 (37.2)	290 (62.8)	
**Sexual behaviours**					
Casual sex*	No	306 (58.5)	57 (18.6)	249 (81.4)	<0.0001
	Yes	217 (41.5)	163 (75.1)	54 (24.9)	
Multiple partner	No	504 (96.2)	202 (40.1)	302 (59.9)	<0.0001
	Yes	20 (3.8)	18 (90.0)	2 (10.0)	
Past STI*	No	445 (84.9)	162 (36.4)	283 (63.6)	<0.0001
	Yes	79 (15.1)	58 (73.4)	21 (26.6)	
Ever treated for STI*	No	448 (85.5)	165 (36.8)	283 (63.2)	<0.0001
	Yes	76 (15.5)	55 (72.4)	21 (27.6)	

* missing values do not make up 524.

### Laboratory findings

In microscopy, about 76% (398) of the samples were positive for Gram negative diplococci (GNDC). In culture 73.1% (383) grew *N*. *gonorrhoea*, 2 (0.4%) grew other organism and 128 (24.4%) did not show any growth.

**Fig 2:**
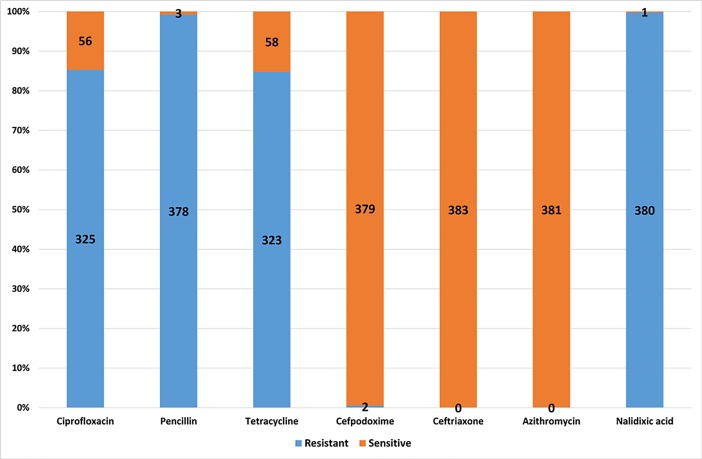
*N*. *gonorrhoeae* antibiotic susceptibility pattern by number and percentages.

The antibiotic susceptibility results showed very high resistance of *N*. *gonorrhoea* against four antibiotics; ciprofloxacin, penicillin, tetracycline and nalidixic acid at 85.1% (325), 99.2% (378), 84.8% (323) and 99.7% (380), respectively. However, sensitivity against cefpodoxime was more than 99.0% (379) and 100% against ceftriaxone and azithromycin (381) (**Fig 2**).

### Inferential analysis

In univariate analysis, the age range of 19–25 were 30% (95% CI 0.46, 0.99) less likely than 26–35 years to be lost to follow up. Armed personnel and others (farmers, diplomats, and unemployed) as compared to civil servant were less likely to lost to follow up at OR 0.3 (95% CI 0.09, 0.85) and OR 0.2 (95% CI 0.45, 0.79) respectively. Persons who had casual sex, multiple sexual partners, never suffered STI or treated for STI in the past were less likely to lost to follow up at OR 0.08, (95% CI 0.05, 0,12), OR 0.07 (95% CI 0.02, 0.32), OR 0.2 (95% CI 0.12, 0.35) and OR 0.2 (95% CI 0.13, 0.38), respectively. However, in multivariate analysis, only those who had casual sex OR 0.1 (95% CI 0.05, 0.14) was significant (**Table 2)**.

**Table 2:** Factors associated with lost to follow up.

**Variable**	**COR**	**95% CI**	**p value**	**AOR**	**95% CI**	**p value**
**Age group (Years)**						
26–35	Ref.					
<19	3.5	0.75, 15.89	0.113	0.8	0.14, 5.13	0.860
19–25	0.7	0.46, 0.99	0.045*	0.7	0.40, 1.10	0.110
36–45	0.7	0.40, 1.28	0.263	0.5	0.26, 1.09	0.080
>45	1.5	0.54, 3.94	0.452	1.4	0.43, 4.37	0.590
**Occupation**						
Civil servant	Ref.					
Private/corporate employee	0.7	0.36, 1.18	0.159	0.7	0.36, 1.50	0.392
Driver	0.7	0.35, 1.51	0.387	1.1	0.46, 2.72	0.813
Student	0.9	0.36, 2.15	0.771	1.3	0.41, 3.91	0.686
Armed personnel	0.3	0.09, 0.85	0.025*	0.4	0.09, 1.62	0.191
Religious boy	0.6	0.18, 1.81	0.342	0.6	0.14, 2.28	0.420
Tourist guide	0.6	0.22, 1.73	0.353	0.4	0.12, 1.47	0.174
Others	0.2	0.45, 0.79	0.022*	0.5	0.09, 2.91	0.457
**Sexual behaviour**						
Casual sex	0.08	0.05, 0.12	<0.001*	0.1	0.05, 0.14	<0.001*
Multiple sexual partner	0.07	0.02, 0.32	0.001*	0.3	0.07, 1.35	0.117
Ever suffered STI	0.2	0.12, 0.35	<0.001*	1.1	0.11, 11.47	0.934
Ever treated for STI	0.2	0.13, 0.38	<0.001*	0.9	0.09, 10.00	0.962

COR, Crude odds ratio; AOR, adjusted odds ratio; CI, confidence interval, p<0.05*

### Treatment and follow up result

Of the 524 patients 66.8% (350) were treated with injection ceftriaxone alone, 32.2% (169) with ceftriaxone and oral doxycycline, 1.0% (5) with ceftriaxone, doxycycline and metronidazole. During follow up only three patients reported persistence of symptoms after treatment. All three had their first samples with a positive culture and a gonococcal isolate sensitive to ceftriaxone, the treatment of choice. Two were treated with injection ceftriaxone alone and one with a combination of ceftriaxone and oral doxycycline. Upon review of the history and clinical examination, one of them did not have any urethral discharge and was considered clinically cured. The other two had continued urethral discharge which grew *N*. *gonorrhoeae* with the same antibiogram on repeat culture. One was a case of re-exposure while the other denied re-exposure and had a probable treatment failure with ceftriaxone and doxycycline dual therapy. The treatment failure occurred despite in-vitro susceptibility to ceftriaxone and he responded to oral azithromycin. Therefore, in this study, probable treatment failure with ceftriaxone was seen only in one patient (0.5%) of the 220 followed up.

## Discussion

This study reports a high rate of clinical cure of gonococcal urethritis patients with the currently recommended treatment regimen in Bhutan. Gonococcal isolates in this study were highly susceptible to the current antibiotics of choice, ceftriaxone. However, there was some gap in the implementation of treatment guidelines resulting into the treatment of very few female patients and non-conformity to the choice of antibiotics as per the national guideline.

In this study, most patients were younger than 36 years belonging to the sexually active group. Therefore, it is important to target this group on safe sex through health education, prevention and control programs. This study recorded very few female patients as found in another recent study from Bhutan [7]. Several factors may have contributed to this finding. Firstly, female patients have higher sub-clinical infections (≥50% asymptomatic) of gonococcal urethritis or cervicitis compared to male patients (≤10% asymptomatic) [2]. Secondly, the non-adherence to the core principles of STI management, namely to counsel, trace and treat the sex partners by treating physicians, failing to trace a female partner for every male patient, might have resulted to fewer female patients. Additionally, STI patients are generally labelled as promiscuous and stigmatized in many communities [19] and the scenario in Bhutan could be worse, being a small and closely-knit society. As a result, people are hesitant to get treatment for STI and this could be more inhibiting to females. From this study, it was obvious that conformity to national guidelines need to be reinforced through sensitization and training programs. Through such programs, it is expected that more patients can get timely and adequate treatment and prevent further spread of the infection. Complications of gonorrhea are more common in females than males [2, 20]. Efforts to increase females to seek treatment through partner tracing could succeed in decreasing the overall burden of STIs and complications associated with it. It can be inferred that patients feel stigmatized because many study participants provided wrong telephone numbers or refused to respond correctly during the follow-up calls. This was one of the main reasons for high attrition rate in this study.

Phuentsholing is the commercial capital of Bhutan and unofficial commercial sex trade is supposedly rampant across the Indian border town of Jaigaon, in West Bengal state [21]. However, a lesser than expected number of patients in PGH could be due to the easy access to private clinics and self-medication in Jaigaon and patients remaining unaccounted. The significantly lesser number of patients lost to follow up in the PGH compared to PGH may also support this conclusion since patients followed up in PGH could be those who had no intention of escaping follow up calls from the investigators. This convenient situation for easy escape across the border to patients makes it difficult for any generalization to study findings. Such circumstances also need to be tackled in the national guidelines and program strategies formulated with a targeted approach to suit local circumstances.

The current Bhutan national antibiotics guideline 2012 recommends only monotherapy with single IM injection of ceftriaxone 250 mg for gonococcal urethritis/cervicitis. However, in this study, 33% (174) patients were treated with a combination of ceftriaxone and other antibiotics such as doxycycline and metronidazole. This non-uniformity in the antibiotic treatment could be due to diverse background of medical graduates joining the Bhutanese health system aggravated by a lack of regular training and orientations on national guidelines to these new recruits. Although treating physicians may justify addition of other antibiotics to cover co-infection with *Chlamydia trachomatis* and other causes of urethritis, this warrants familiarization and training of physicians on the existing national guidelines for uniform treatment to prevent antibiotic overuse.

The resistance of *N*. *gonorrhoeae* in Bhutan against ceftriaxone, the current therapy of choice, is not as alarming as in some other countries. This low resistance could probably be due to less antibiotic uses because of the regulated sale of antibiotics without prescription, no private medical clinics and limited opportunities for self-treatment. However, antibiotic resistance in gonococci is an emerging issue globally and there should be a constant monitoring. In addition, with increased Bhutanese travelling outside and Bhutan emerging as a travel destination, there is a risk of importing highly resistant gonococci into the country. With both the WHO and the CDC recommending dual therapy or monotherapy supported by local data for urogenital infections, continued vigilance will prompt any need for a change of the choices of empirical treatment. Antibiotic resistance surveillance should be scaled up to more hospitals since currently only four of the 28 national hospitals have microbiology culture facilities. The current use of disc diffusion method for susceptibility testing should be replaced with the more reliable method of MIC testing using commercial E-tests. The reported single case of probable treatment failure with ceftriaxone and doxycycline in this study should be interpreted with caution as the inference was based solely on patient’s claim of not being re-exposed which may be unreliable due to the sensitive nature of the infection.

This study has a few important limitations. Since only two hospitals were included in the study, the data is poorly representative of the country. The inclusion criteria was generalized and included all urethritis suspects and definition of cure was overtly clinical with no microbiological evidence of cure. While the use of quantitative MIC is the current trend where available, the data in this study was completely based on the disc diffusion method and may be comparable only with regional countries that use the same method. As patient follow-up was solely based on telephone calls with a significant number of patients lost to follow-up, the high percentage of cure reported need confirmation in future studies with vigorous follow-up including microbiological test of cure.

## Conclusions

This study demonstrated low resistance of *N*. *gonorrhoeae* to currently recommended antibiotics and favorable treatment outcomes in Bhutan. A continued surveillance of antimicrobial resistance to *N*. *gonorrhea* including more hospitals is important to monitor the emergence of antibiotic resistance. It is recommended that STI treatment guidelines need to be implemented strictly; focusing on correct treatment regimen, partner treatment and addressing the social stigma associated to STI. National agencies should align their programs towards more women-friendly centres for effective prevention, control and management of STIs. Future studies with more number of patients throughout the country should consider both clinical and microbiological test of cure for a more dynamic finding.

## Supporting information

S1 Table Antibiotic disc strengths, annular radius break points and interpretative criteria for N. gonorrhoeae by CDS method. (DOCX)

S1 Metadata Metadata of the study. (XLSX)
