# Supplementary material for: Antibiotic resistance in Neisseria gonorrhoea and treatment outcomes of gonococcal urethritis suspected patients in two large hospitals in Bhutan, 2015
Source: PLoS One. 2018 Aug 1;13(8):e0201721. doi: 10.1371/journal.pone.0201721 (PMC6070275; doi:10.1371/journal.pone.0201721)
Supplement: S1 Table — (DOCX) [file pone.0201721.s001.docx]

**S1 Table: Antibiotic disc strengths, annular radius break points and interpretative criteria for *N. gonorrhoeae* by CDS method**

| Antibiotics | Disc strength | Interpretation criteria: Annular radius | | | |
| --- | --- | --- | --- | --- | --- |
|  |  | **S** | **DS/LS** | **R** | **Remarks** |
| Penicillin | 0.5U | >9 mm | 3-9 mm | <3 mm |  |
| Ciprofloxacin | 1 µg | >11 mm | 6-11mm | ≤6 mm | Tested together and interpreted with annular diameter of both the antibiotic discs |
| Nalidixic acid | 30 µg | >6 mm | ≤6 mm | ≤6 mm |  |
| Spectinomycin | 100 µg | ≥ 6mm | - | <6 mm |  |
| Tetracycline | 10 µg | ≥2mm  (Not TRNG) | - | ≤ 1mm (TRNG) |  |
| Azithromycin | 15 µg | > 6mm | - | ≤6mm |  |
| Ceftriaxone | 0.5 µg | >9 mm | 5-9 mm | - | Annular radius of ≤9mm require MIC determination for a definite categorization of susceptibility |
| Cefpodoxime | 10 µg | >12mm | ≤12mm | - |  |

S, susceptible; DS, decreased susceptibility; LS, less susceptible; R, Resistant; TRNG, tetracycline-resistant *N. gonorrhoeae*
